# Drotrecogin alfa (activated) may attenuate severe sepsis-associated encephalopathy in clinical septic shock

**DOI:** 10.1186/cc8947

**Published:** 2010-04-07

**Authors:** Herbert Spapen, Duc Nam Nguyen, Joris Troubleyn, Luc Huyghens, Johan Schiettecatte

**Affiliations:** 1 Intensive Care Department, University Hospital, Vrije Universiteit Brussel, Laarbeeklaan 101, B-1090 Brussels, Belgium; 2 Department of Immunochemistry, University Hospital, Vrije Universiteit Brussel, Laarbeeklaan 101, B-1090 Brussels, Belgium

## Abstract

**Introduction:**

Sepsis-associated encephalopathy (SAE) is a diffuse cerebral dysfunction induced by the immuno-inflammatory response to infection. Elevated levels of the brain-specific S100B protein are present in many septic patients and reflect the severity of SAE. Adjunctive treatment with drotrecogin alfa (activated) (DrotAA), the human recombinant form of activated protein C, has been shown to improve mortality in patients with severe sepsis-induced organ failure. We studied the effect of DrotAA on S100B levels in patients with acute septic shock who presented with increased baseline values of this biomarker.

**Methods:**

All patients received standard goal-directed resuscitation treatment. Patients with pre-existing or acute neurological disorders were excluded. Based on the Glasgow coma scale (GCS), patients were classified into two groups: GCS ≥ 13 and GCS <13. DrotAA was given as a continuous infusion of 24 μg/kg/h for 96 h. S100B was measured before sedation and the start of DrotAA (0 h) and at 32 h, 64 h and 96 h and at corresponding time points in patients not treated with DrotAA. The lower limit of normal was < 0.5 μg/L.

**Results:**

Fifty-four patients completed the study. S100B was increased in 29 (54%) patients. Twenty-four patients (9 with GCS ≥ 13 and 15 with GCS <13) received DrotAA. S100B levels in DrotAA-treated patients with a GCS <13, though higher at baseline than in untreated subjects (1.21 ± 0.22 μg/L vs. 0.95 ± 0.12 μg/L; *P *= 0.07), progressively and significantly decreased during infusion (0.96 ± 0.22 μg/L at 32 h, *P *= 0.3; 0.73 ± 0.12 μg/L at 64 h, *P *< 0.05; and 0.70 ± 0.13 μg/L at 96 h, *P *< 0.05 vs. baseline). This patient group had also significantly lower S100B values at 64 h and at 96 h than their untreated counterparts. In the patients with a GCS ≥ 13, S100B levels were not influenced by DrotAA treatment.

**Conclusions:**

S100B-positivity is present in more than half of the patients with septic shock. When increased S100B levels are used as a surrogate for SAE, adjunctive DrotAA treatment seems to beneficially affect the evolution of severe SAE as discriminated by an admission GCS <13.

## Introduction

Sepsis-associated encephalopathy (SAE) is a diffuse cerebral dysfunction accompanying an evolving septic state. The pathophysiological alterations underlying SAE are incompletely understood. Basically, there is no direct infection of the brain. Rather, both inflammatory and non-inflammatory processes are involved that induce blood-brain barrier breakdown, cerebrovascular endothelial activation, deregulation of brain metabolic pathways, and brain cell apoptosis [1].

Activated protein C (APC) plays a key role in the preservation of endothelial function and microvascular perfusion during severe sepsis and septic shock. Hence, a dysfunctional protein C pathway is thought to contribute largely to sepsis-induced microvascular and subsequent organ failure. Drotrecogin alfa (activated) (DrotAA), a recombinant human APC, has been shown to improve the microcirculation *in vivo*. DrotAA also binds directly to specific receptors on endothelial and inflammatory cells, thereby modulating and downregulating inflammatory and apoptotic processes [2]. Adjuvant therapy with DrotAA has been shown to significantly reduce mortality in adult patients with severe sepsis [3]. Survival rate was highest in the most severely ill patients and largely driven by a more rapid improvement of cardiovascular and respiratory failure [4].

We have previously demonstrated that levels of the highly brain-specific protein S100B were increased in low consciousness SAE, suggesting a possible use for this protein as a biomarker of SAE [5]. We investigated whether and how S100B serum levels, when used as a biochemical surrogate for SAE, were influenced when DrotAA was added to standard treatment in patients with septic shock.

## Materials and methods

The study was approved by the Ethics Committee of our hospital and was conducted in compliance with the Declaration of Helsinki. Patients with pneumonia-induced septic shock were included after informed consent was obtained from a next of kin. Pneumonia was either acute-onset community-acquired or nosocomial and characterized by bilateral infiltrates on chest x-ray and a PaO_2_/FiO_2 _ratio <300. Septic shock was defined as sepsis-induced hypotension along with the presence of perfusion abnormalities, initially not responding to adequate fluid resuscitation. Patients were excluded when one of the following criteria was present: age <18 years; pregnancy or nursing state; renal and hepatic failure; primary central nervous disorders (for example, meningitis, neoplasm, stroke, head injury, known epilepsy); peripheral or critical illness polyneuropathy; alcohol or drug abuse; Wernicke encephalopathy; acute mental deterioration secondary to non-septic metabolic disorders with organ dysfunction; sepsis associated with dismal prognosis and imminent death; and sepsis occurring within two weeks after cardiac resuscitation, severe burns, trauma, orthopaedic surgery, cardiac bypass surgery, or neurosurgery.

Resuscitation aimed to obtain and maintain a mean arterial blood pressure ≥ 70 mmHg, a S_cv_O_2 _>70% and a correct cardiac output (confirmed by transoesophageal echocardiography). To achieve these goals, all patients received colloid and crystalloid volume suppletion and, if needed, dobutamine (up to 20 μg/kg/minute) and/or norepinephrine (up to 1.5 μg/kg/minute). All patients were mechanically ventilated under continuous infusion with a combination of propofol (up to 12 mg/kg/h) or midazolam (up to 0.3 mg/kg/h) and fentanyl (up to 0.05 mg/kg/minute). Cisatracurium (1 to 2 mg/kg/minute) was added to obtain adequate patient-ventilator synchronization when necessary. Patients were treated with empirical broad-spectrum antibiotic therapy, which was adjusted according to culture results and received stress doses of hydrocortisone (100 mg loading dose followed by a continuous infusion of 0.18 mg/kg/h). Insulin was infused in all patients to keep glycaemia between 100 and 150 mg/dL. Patients were not randomized whether or not to receive DrotAA. Prescription of DrotAA followed national guidelines but the decision to start the drug was left at the discretion of the attending physician. When DrotAA was given, it was administered as a 24-h continuous infusion of 24 μg/kg/minute during four consecutive days.

In all patients, the Glasgow Coma Scale (GCS) was assessed before tracheal intubation and start of sedation. Patients were divided into two groups according to this *baseline *GCS being either >13 or <13. A GCS cut-off at 13 was chosen based on the observations of Eidelman et al. who found a three-fold increase in mortality when the GCS decreased below 13 [6]. Patients with a GCS <13 underwent a contrast computed brain tomography (Siemens, Sensation 16 Multislice, Forchheim, Germany) to exclude pre-existing organic or vascular cerebral lesions. A lumbar puncture was performed in all patients with a GCS ≤ 8 to exclude brain or meningeal infection.

Blood was drawn from a radial arterial catheter for measurement of S100B before the start of DrotAA infusion (0 h) and at three distinct time points (32 h, 64 h, and 96 h) thereafter. In patients who did not receive DrotAA, blood for S100B determination was taken at corresponding time points. All samples were immediately centrifuged and aliquoted at -70°C until analysis. S100B was measured with a monoclonal two-site immunoradiometric assay to detect the S100B subunit (Sangtec 100, Sangtec Medical AB, Dietzenbach, Germany). Test imprecision between days was <6%, and the lower limit of normal value was < 0.5 μg/L.

The study was discontinued in patients who developed significant renal impairment (defined as a two-fold increase of baseline serum creatinine or the need to start renal replacement therapy) during the study period. Patients who survived at least 96 h following enrolment in the study were evaluated. ICU and hospital mortality were defined as being alive respectively at ICU discharge and at the end of hospital stay.

SPSS package version 13.0 for Windows (SPSS Inc, Chicago, IL, USA) was used for statistical analysis. Chi-square and Student's *t- *test were used to evaluate differences in age, gender, mortality and Sequential Organ Failure Assessment (SOFA) and Acute Physiology and Chronic Health Evaluation (APACHE) II scores between patients with GCS ≥ and <13. S100B levels between both GCS groups were compared using a one-way analysis of variance for repeated measurements followed by Bonferroni test for multiple comparisons. Data were expressed as means ± SD or means ± SEM. Statistical significance was accepted at *P *< 0.05.

## Results

Fifty-four patients (33 men; 21 women) completed the study protocol. At study entry, 23 had a GCS ≥ 13 and 31 had a GCS <13. S100B levels were elevated in 29 (54%) patients and exceeded 1 μg/L for at least one measurement in 12 (22%) patients. Sixty-five percent of patients with a GCS <13 and 45% of patients with a GCS ≥ 13 had increased S100B levels.

Patients with a GCS <13 had significantly higher APACHE II scores, baseline SOFA scores and higher baseline S100B values than those with a GCS ≥ 13 (Table 1).

**Table 1:** Patient characteristics and mortality

	GCS ≥ 13	GCS <13
Gender (M/F; n)	12/11	21/10
Age (years; mean ± SD)	69 ± 13	72 ± 10
APACHE II score (mean ± SD)	17 ± 4 *	25 ± 7
Baseline SOFA score (mean ± SD)	8 ± 2 *	10 ± 3
Baseline S100B (μg/L; mean ± SEM)	0.70 ± 0.08°	0.96 ± 0.15
ICU mortality (n; %)^†^		
DrotAA	6 (67)	10 (67)
No DrotAA	7 (50)	9 (56)
Hospital mortality (n; %)		
DrotAA	6 (67)	13 (86)
No DrotAA	10 (71)	12 (75)

* *P *< 0.05, °*P *= 0.05 as compared to patients with GCS <13

^† ^number and percentage of DrotAA-treated or -untreated patients who died

APACHE: Acute Physiology and Chronic Health Evaluation; DrotAA: drotrecogin alfa (activated); F: female; ICU: intensive care unit; M: male; SOFA: Sequential Organ Failure Assessment

**Figure 1:**
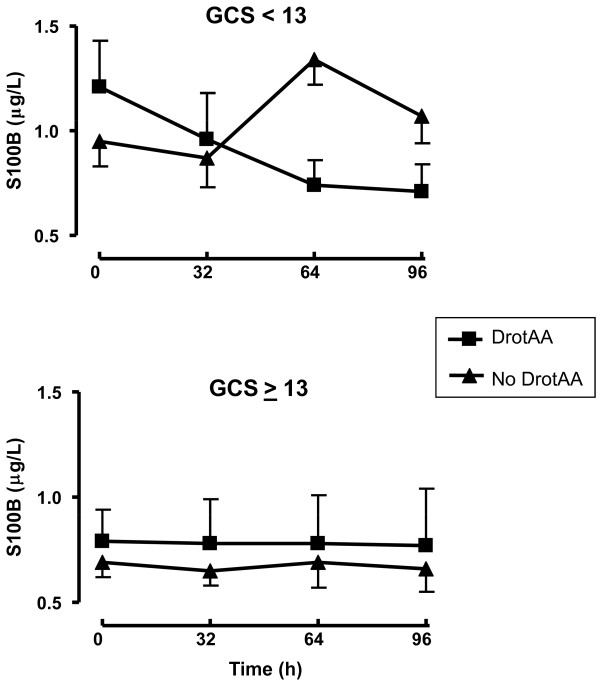
**S100B levels in patients with GCS <13 and GCS ≥ 13, with or without DrotAA treatment**. * *P *< 0.05 as compared to baseline S100B levels in DrotAA-treated patients; °*P *< 0.05 DrotAA-treated vs. -untreated patients. Values are means ± SEM DrotAA: drotrecogin alfa (activated); GCS: Glasgow Coma Scale; SEM: standard error of the mean.

Twenty-four patients (9 with GCS ≥ 13 and 15 with GCS <13) received DrotAA. In the group of patients with a GCS <13, those who received DrotAA tended to have higher baseline S100B levels than their untreated counterparts (1.21 ± 0.22 vs 0.95 ± 0.12 μg/L; *P *= 0.07). S100B levels progressively decreased during DrotAA infusion in patients with a GCS <13 only (0.96 ± 0.22 μg/L at 32 h; 0.73 ± 0.12 μg/L at 64 h; 0.70 ± 0.13 μg/L at 96 h; all means ± standard error of the mean (SEM); *P *respectively = 0.3; < 0.05; and < 0.05 compared to baseline). Compared to untreated patients, those who received DrotAA, had significantly lower S100B values at 64 h (1.34 ± 0.12 vs. 0.73 ± 0.12 μg/L; *P *< 0.05) and at 96 h (1.07 ± 0.13 vs. 0.70 ± 0.13 μg/L; *P *< 0.05) (Figure 1, upper panel). In contrast, patients with a GCS ≥ 13, had comparable S100B levels within and between groups, that were not affected by DrotAA treatment (Figure 1, lower panel).

Global ICU and in-hospital mortality were high (respectively 59% and 76%) and did not differ between DrotAA-treated and -untreated patients (ICU mortality 62.5% vs. 57%; hospital mortality 75% vs. 77%; both *P *> 0.05). Mortality was also not different between patients with GCS values above or below 13, regardless DrotAA was given or not (Table 1).

## Discussion

Patients with septic shock frequently have alterations in consciousness ranging from mild stupor to coma which cannot be attributed to brain lesions, haemodynamic instability or metabolic disorders [6]. This so-called SAE remains often unnoticed within the clinical spectrum of septic shock that is mostly dominated by life-threatening cardiovascular, respiratory, and renal complications. However, SAE is to be considered as a distinct sepsis-induced organ dysfunction since it is characterized by local expression of pro-inflammatory cytokines in the absence of gross abnormalities of cerebral blood flow or direct infectious involvement of the brain. The pathophysiology of SAE is multifactorial and features cerebrovascular endothelial dysfunction, blood-brain barrier disruption and abnormal neurotransmitter patterns. Main pathological findings include haemorrhagic lesions, microthrombi and abscesses, cyto- and vasogenic oedema, and multifocal necrotizing leukoencephalopathy [1,7,8]. Also, neuronal and microglial apoptosis is detected in brain areas that are involved in the neuro-endocrine and behavioural response to stress [9].

Bedside diagnosis and follow-up of SAE are cumbersome. Clinical neurological evaluation, essentially based on GCS assessment, is difficult and rapidly becomes futile when analgesic sedation is started. Daily interruption of sedation is often not feasible during treatment of pneumonia-induced septic shock and may also confound SAE with sedation withdrawal effects. Electroencephalography (EEG) is a more sensitive method to detect brain dysfunction. However, EEG patterns in sepsis are hampered by sedation, show a broad range of interindividual variability, and are difficult to quantify. Also, S100B levels do not correlate with either the GCS or EEG patterns in septic patients [10]. The recording of somatosensory evoked potentials (SEP) provides an elegant and reliable estimation of the severity of SAE [11]. Still, this technique is difficult to use in routine and the relationship between the degree of SEP impairment and corresponding S100B levels in SAE remains to be determined. Cerebrospinal fluid assay and neuroimaging cannot be considered monitoring tools. With the exception of specific situations (seizures, focal neurological signs, suspicion of meningeal or cerebral infection), it is also impossible to correctly time these interventions during the course of illness.

S100B is a low-molecular weight, calcium-binding protein secreted by glial and Schwann cells. S100B is released following brain injury of various aetiology. Serum levels of this biomarker correlate positively with the degree of brain injury and neuronal apoptosis and predict the outcome [12-14]. We have previously shown that S100B levels in severe sepsis better reflected the presence and prognosis of low consciousness SAE than the GCS. S100B levels were increased in 42% of patients, exceeding 1 μg/L in 11%. S100B levels between 0.06 and 2 μg/L were typically associated with white matter lesions which are thought to represent the pathological substrate of SAE [5]. In the present study, a higher incidence of S100B elevation was found which probably relates to a higher severity of illness in the patient population studied. The observed high mortality rate is also in line with our earlier findings, indicating a 70% mortality in S100B-positive patients with severe sepsis [5]. It could be argued that the observed high S100B levels might be, at least partially, of extracerebral origin. This is unlikely since common sources of S100B release such as renal failure and surgical tissue injury were either excluded or avoided. Whether serum S100B elevation in sepsis always reflects blood-brain barrier disruption is difficult to prove. Data are conflicting with some authors demonstrating high levels of S100B [15] while others report no evidence of S100B increase [16] in the cerebrospinal fluid. We were unable to measure S100B in the cerebrospinal fluid because lumbar puncture is an absolute contraindication during DrotAA infusion due to the bleeding risk.

A specific treatment for SAE does not exist. The outcome of SAE is considered to be dependent on prompt and appropriate treatment of the septic process as a whole. Besides control of infection, this also implies management of organ failure, correction of metabolic disturbances, and avoidance of neurotoxic drugs. There is no clinical evidence that adjuvant therapy such as strict glycaemic control with insulin, stress doses of steroids or APC either reduce the incidence or influence the severity and evolution of SAE. Insulin may be neuroprotective as it can prevent hyperglycaemia-induced oxidative stress and apoptotic cell death. However, anticipating insulin effects on SAE is impossible due to the complexity of cerebral glucose metabolism and the high variability in glucose levels between patients. Moreover, intensive insulin therapy does not improve outcome of patients with ischaemic [17] or traumatic [18] brain injury and significantly increases the risk of hypoglycaemia which, by itself, can induce neurocognitive dysfunction [19]. Steroids can decrease systemic inflammation and high doses are known to reduce brain oedema and to restore blood brain barrier function [20]. However, high-dose steroids are associated with higher mortality and increase the risk for secondary infection and hepatorenal dysfunction [21,22]. Stress doses, as given in our patients, have not been shown to reduce serum S100B in septic shock [23].

APC, commercialized as DrotAA, is approved by both the American and European Drug Agencies as an adjunctive treatment of severe sepsis in patients with organ failure and/or at high risk of death. Independent of its clinically apparent anticoagulant activity, APC directly interferes at the interface between the (micro)vascular endothelium and the innate immune response. The binding of APC on endothelial and inflammatory cell receptors exerts pleiotropic *in vitro *intra- and intercellular effects, altering gene expression profiles, inhibiting apoptosis and down-regulating inflammation. As a result, APC preserves, protects and probably even restores endothelial function which may attenuate ongoing and prevent further organ damage [24]. This concept is corroborated by the proven benefit of adjunctive DrotAA treatment on cardiovascular, respiratory and haematological disorders in severe clinical sepsis [4]. The effect of the drug on sepsis-induced renal and cerebral dysfunction, however, is less evident, mainly because sensitive parameters for objective evaluation of these organ systems are lacking.

The cerebral effects of APC become progressively elucidated. In mice, APC crosses the blood-brain barrier via binding on the endothelial protein C receptor, acts directly on neurons, microglial cells, microvessels and motor neurons [25], and protects stressed brain endothelial cells from hypoxic/ischaemic damage [26]. In a rat model of periventricular leukomalacia, an injury that mimics the pathophysiological findings of SAE, treatment with APC was associated with less endotoxin-induced white matter and myelination deficits. This protective effect was associated with a decrease in neuronal apoptosis and reduced local expression of pro-inflammatory cytokines [27]. Finally, APC has neuroprotective activity in human ischaemic brain [28] by regulating cytosolic Ca^2^^+ ^flux and blocking apoptosis in brain endothelial cells [29]. The present study suggests that APC may attenuate SAE but only in patients with clinically more severe neurological dysfunction. This is in line with previous observations showing that APC tends to be more effective in the most critically ill patients.

Our study has important limitations. Though representing a rather homogenous septic shock population, the number of patients is small. This precludes a reliable assessment of any possible impact of structural components of the sepsis resuscitation protocol (amount and type of fluid, catecholamine use, sedation level, glycaemic control, nutritional status) on the evolution of SAE. Patients were also studied for a short period of time and S100B behaviour after DrotAA treatment had been stopped is unknown. The decrease in S100B in DrotAA-treated patients was not paralleled by a decrease in mortality. This suggests that the presumed attenuation of SAE does not play a preponderant role in septic shock survival.

## Conclusions

This study is the first to show a probable beneficial effect of APC on the evolution of severe SAE, defined as an admission GCS <13, in clinical septic shock. The usefulness of S100B as a serum biomarker for bedside diagnosis of SAE and its role in the evaluation of specific treatment effects on this particular condition merits further investigation. Our findings also call for more comprehensive experimental and clinical research to clarify the relationship between S100B protein behaviour and the extent of concomitant electrophysiological, cerebrovascular, and neurohormonal alterations in severe clinical sepsis and septic shock.

## Key messages

▪ The S100B peptide is a potential serum biomarker for bedside diagnosis and follow-up of sepsis-associated encephalopathy.

▪ Adjunctive treatment with activated protein C may attenuate the encephalopathy that accompanies septic shock. However, the beneficial effect is only present in more severe encephalopathy and does not affect mortality.

## Abbreviations

APACHE: Acute Physiology and Chronic Health Evaluation; APC: activated protein C; DrotAA: Drotrecogin alfa (activated); EEG: electroencephalography; GCS: Glasgow Coma Scale; ICU: intensive care unit; SAE: sepsis-associated encephalopathy; SEP: somatosensory evoked potentials; SOFA: Sequential Organ Failure Assessment.

## Competing interests

HS has been an investigator for studies sponsored by Eli-Lilly, the manufacturer of drotrecogin alfa (activated), and has received an honorarium from Eli-Lilly for serving as a member of local Advisory Boards. All other authors declare that they have no competing interests.

## Authors' contributions

HS and DNN conceived the study, and analyzed, interpreted and integrated the data. They also elaborated the manuscript. JT and LH performed and supervised the enrolment of patients in the study. JS performed the S100B assay. All authors read and approved the final version of the manuscript.
